# Identification of TIFY Family Genes and Analysis of Their Expression Profiles in Response to Phytohormone Treatments and *Melampsora larici-populina* Infection in Poplar

**DOI:** 10.3389/fpls.2017.00493

**Published:** 2017-04-05

**Authors:** Wenxiu Xia, Hongyan Yu, Pei Cao, Jie Luo, Nian Wang

**Affiliations:** College of Horticulture and Forestry Sciences, Huazhong Agricultural UniversityWuhan, China

**Keywords:** TIFY domain gene family, poplar, jasmonic acid, salicylic acid, gene expression, *Melampsora larici-populina*

## Abstract

The TIFY domain contains approximately 36 conserved amino acids that form the core motif TIF[F/Y]XG, and they were reported to play important roles in plant growth, tissue development and defense regulation. Moreover, more and more evidence has shown that some members of the TIFY gene family perform their functions by modulating plant hormone signaling pathways. Poplar trees are found worldwide, and they comprise approximately 30 species. Benefit from the importance of poplar and its advanced platform, this tree is considered to be the model perennial plant. Here, we conducted a genome-wide identification of TIFY genes in poplar, and 24 TIFY genes were found. These 24 TIFY genes were assigned to different subfamilies according to the presence or absence of domains and motifs that they harbored. Careful analyses of their locations, structures, evolution and duplication patterns revealed an overview of this gene family in poplar. The expression profiles of these 24 TIFY genes were then analyzed in different tissues using publicly available expression data; their expression profiles following different JA/SA treatments and infection with leaf rust pathogen were also carefully examined by qRT-PCR assays. Based on their expression profiles, the functions of a number of TIFY genes could be predicted. By performing this study, we have provided valuable information for further functional characterisation of TIFY genes in poplar and candidate genes for the improvement of poplar disease resistance.

## Introduction

The TIFY domain contains approximately 36 conserved amino acids (AA) that form the core motif TIF[F/Y]XG (Vanholme et al., 2007; Bai et al., 2011). This conserved domain characterizes a plant-specific family of transcription factor (TF) genes called the TIFY gene family. TIFY genes were first characterized in Arabidopsis, and the gene *AT4G24470* was reported to be a putative TF involved in inflorescence and flower development (Nishii et al., 2000). However, this gene was annotated as a ZIM gene in this study (Nishii et al., 2000). With a genome-wide survey of the Arabidopsis genome and because of the confusing use of the ZIM nomenclature, proteins containing TIFY domains were renamed as the TIFY gene family (Vanholme et al., 2007). The TIFY gene family can be classified into four subfamilies, TIFY, JAZ, ZML, and PPD, depending on whether they contain additional domains/motifs (Vanholme et al., 2007; Bai et al., 2011). Proteins with only the TIFY (PF06200) domain are classified as the TIFY subfamily; proteins with both the TIFY and jasmonate ZIM domains (JAZ, PF09425) are classified as the JAZ subfamily (Staswick, 2008); and proteins containing the TIFY domain and the CCT (PF06203) and/or ZML (PF00320) motif are classified as the ZML subfamily. In the PPD subfamily, the proteins contain the TIFY and PPD domains; proteins in this subfamily also sometimes contain a truncated JAZ domain (White, 2006).

To date, the functions of several TIFY genes have been fully investigated, and some of them have been found to play important roles in different biological processes. Of the four TIFY subfamilies, the functions of JAZ proteins are the most clear, and they have been found to be involved in the jasmonate (JA) signaling pathway. JAZ proteins act as JA repressors to inhibit TFs that regulate early JA-responsive genes (Pauwels and Goossens, 2011); in contrast, JA can also induce degradation of JAZ proteins, thereby allowing the expression of its response genes (Chung and Howe, 2009; Niu et al., 2011). Since JA is a key phytohormone in plant development, JAZ proteins were also found to play critical roles in the regulation of numerous aspects of plant development. In *Astragalus sinicus*, AsJAZ1 was found to interact with AsB2510 and participated in nodule development and nitrogen fixation (Li et al., 2015). In Arabidopsis, deletion of the two PPD genes (at the same locus) increased leaf lamina size and resulted in dome-shaped rather than flat leaves. Siliques were also altered in shape because of the additional lamina growth (White, 2006). JAZ and DELLA proteins were also found to bind to the WD-repeat/bHLH/MYB complex to modulate the synergistic effects of gibberellin and JA signaling; thus, these two types of proteins can integrate different hormonal signals to synergistically regulate plant development (Qi et al., 2014). Moreover, JAZ proteins were also found to play important roles in plant defense. In wild soybean, transcription of *GsJAZ2* increased following exposure to different abiotic stresses including salt, alkali, cold and drought. Overexpression of *GsJAZ2* in an Arabidopsis line resulted in enhanced tolerance to salt and alkali stresses (Zhu et al., 2012). In rice, OsJAZ8 was reported to regulate host immunity by modulation of JA-responsive volatile compounds (Taniguchi et al., 2014). In addition to the JAZ genes, other genes of the TIFY subfamilies were found to play roles in plant development and defense. In wild soybean, GsTIFY10, which could be induced by bicarbonate, salinity stress and JA, was isolated and overexpressed in Arabidopsis. The transgenic plants showed enhanced tolerance to bicarbonate stress during seed germination and during the early- and adult-seedling developmental stages (Zhu et al., 2011). Based on the preceding information, we conclude that the plant-specific TIFY proteins are very important in the regulation of plant development and defense.

Due to the importance of the TIFY gene family and benefitting from the accelerated release of genome data, a number of studies have focused on the genome-wide investigation and characterisation of TIFY genes in different plant species. In the respective dicot and monocot model plants, Arabidopsis and rice, 18 and 20 TIFY genes were reported, respectively (Ye et al., 2009; Bai et al., 2011). In addition, 18 TIFY family proteins were found in pigeonpea [*Cajanus cajan* (L.) Millsp.] (Sirhindi et al., 2016); 30 TIFY genes were found in apple (*Malus* × *domestica* Borkh.) (Li et al., 2014); 19 TIFY genes were found in grape (*Vitis vinifera*) (Zhang et al., 2012); 28 TIFY genes were found in the *Gossypium raimondii* genome (He et al., 2015); 21 TIFY genes were found in *Brachypodium distachyon* (Zhang et al., 2015); and 34 TIFY genes in found in wild soybean (*Glycine soja*) (Zhu et al., 2013). In these studies, the expression profiles of TIFY genes were investigated and their functions were predicted based on their preferentially transcriptional abundance in a given tissue or under a given stress condition. This information is very valuable for guiding further characterisation of the functions of TIFY genes. In poplar, 25 TIFY proteins were identified using the *Populus trichocarpa* genome version 1.0 annotations when global comparisons of the gene family were performed in a number of plant species (Bai et al., 2011). Recently, the *P. trichocarpa* genome annotation was updated to version 3.0, and the quality of the reference genomic sequences was also greatly improved. Therefore, it is necessary to conduct a genome-wide survey of TIFY genes in poplar using the *P. trichocarpa* genome annotation version 3.0, in addition to analyzing their expression profiles under different conditions.

Poplar (*Populus* spp.) is a tree that is found worldwide, and it comprises approximately 30 species. Due to its characteristics of being fast-growing, widely used and strong ability to adapt, poplar is grown in most of the Northern hemisphere. Most of the species in the genus can be used for wood, pulp, paper, and fuel. In some areas, poplar is also an important landscape tree. Moreover, poplar species usually have small genome sizes, and some species can easily be created transgenic lines. In 2006, the draft genomic sequence of *P. trichocarpa* was released (Tuskan et al., 2006). Subsequently, the genomic sequence and annotations have been updated several times and high quality genomic information has recently become available online^1^ (Wullschleger et al., 2013). Moreover, additional genomic information of value for the species is publicly available, including the expression database and the whole genomic sequence of *P. euphratica* (Ma et al., 2013; Sundell et al., 2015). According to the above information, poplar is considered to be a model plant in perennial tree species.

To our knowledge, no comprehensive genome-wide survey and characterisation of TIFY genes in poplar has been carried out. In this study, we mainly focused on the responses of TIFY genes to phytohormone treatments and biotic stresses, and we aimed to identify TIFY genes involved in poplar biotic defenses. To this end, we first conducted a genome-wide identification of TIFY genes using the *P. trichocarpa* genome annotation version 3.0; 24 TIFY genes were found. Based on the presence of certain domains/motifs within these 24 TIFY genes, they were assigned to different subfamilies. The duplication types of each of the TIFY genes were simulated and their phylogeny, gene structures and locations within the genome were also investigated. Based on the publicly available expression data, the expression profiles of the 24 TIFY genes in different tissues were subsequently analyzed; their expression profiles after different JA/SA treatments and *M. larici-populina* infection were also carefully examined by qRT-PCR assays. Based on their expression profiles, the functions of a number of TIFY genes could be predicted. By performing this study, we have provided valuable information for further functional characterisation of TIFY genes in poplar and candidate genes for the improvement of poplar disease resistance.

## Materials and Methods

### Identification of TIFY Genes in the Poplar Genome

To identify members of the TIFY gene family in poplar, the Hidden Markov Model (HMM) profile of the TIFY domain (PF06200) was downloaded from the pfam database (Pfam^2^). The reference genome *P. trichocarpa* was downloaded from the Phytozome database^3^. Genome annotation version 3.0 of *P. trichocarpa* was used in this study. To search the 73,013 predicted proteins of the *P. trichocarpa* genome annotation version 3.0, 759 seed sequences in the PF06200 HMM profile were used. Two softwares, blastp and HMMER, were used to perform a search for proteins harboring the TIFY domain. Proteins of *P. trichocarpa* that showed *E*-values above 1e-6 in the search results of blastp or HMMER were considered to be candidate TIFY domain genes. Of the proteins from the same gene model, only the longest ones (the “0.1” gene model of TIFY genes in the *P. trichocarpa* genome annotation version 3.0) were kept for further study. To confirm the existence of the TIFY domain and the Jas, CCT and ZML motifs, candidate TIFY domain proteins were used to search the Pfam database. The Pfam accession numbers for the three motifs are PF09425, PF06203 and PF00320, respectively. We also checked for the existence of the PPD domain/motif in each of the candidate TIFY genes. We noted that there is no sequence information for the PPD domain/motif in the Pfam database. The previously annotated PPD genes in Arabidopsis and grape were used to build the conserved PPD sequence. Briefly, the previously annotated PPD sequences were first aligned by ClustX2.1, and an msf result was produced and imported into hmmbuild implemented in HMMER. The resulting PPD HMM profile was used to search all of the candidate TIFY genes.

### Analyses of Phylogeny, Genomic Structures, Chromosomal Locations and Gene Duplications

The software MEGA5 was used to analyze the phylogeny of all of the TIFY genes (Tamura et al., 2011). The protein sequences of all of the identified poplar TIFY genes were imported into ClustalX2.1 to perform a complete alignment, and the resultant multiple alignment file was imported into MEGA5. Unrooted phylogenetic trees were constructed using the Neighbor-Joining (NJ) method, and the bootstrap test was carried out with 1,000 iterations. For constructing phylogenetic trees among the four species, the protein sequences of the three other species were obtained from Phytozome database^4^. The deduced TIFY protein information was obtained from *Arabidopsis thaliana* (Bai et al., 2011), *Oryza sativa* (Bai et al., 2011) and *Vitis vinifera* (Zhang et al., 2012).

Gene structures and their chromosomal locations were obtained from the *P. trichocarpa* genome annotation version 3.0. The gene structures were displayed using a custom R script. The duplication pattern for all of the poplar genes was analyzed using *MCScanX* software. Briefly, the 41,335 poplar gene models (the “0.1” gene model in the *P. trichocarpa* genome annotation version 3.0 represents all genes models if a gene has more than one alternative transcript) were extracted from the poplar genome. An all-vs.-all local blast for the 41,335 poplar gene models using the local blast software with *E*-values under 1e-4 was carried out. The blast output was imported into *MCScanX* software following the procedure. All of the 41,335 genes were classified into four types using default criteria, including segmental, tandem, proximal, and dispersed duplications. The duplication pattern for each poplar *TIFY* gene was then determined according to the results.

### Microarray-Based Expression Analysis of Poplar TIFY Genes Using Publicly Available Data

The *PopGenExpress* database was employed to investigate the expression levels of all of the poplar *TIFY* genes in different tissues^5^ (Wilkins et al., 2009). Briefly, the correspondence between microarray probes and poplar genes was obtained from the annotation files deposited for the microarray data. Because the annotation released for the poplar microarray used in the *PopGenExpress* database was based on *P. trichocarpa* genome annotation version 2.0, the relationship between the probes and poplar genes annotated in genome version 3.0 was transformed based on the correspondence between versions 2.0 and 3.0 of the *P. trichocarpa* genome annotation. For genes that had no corresponding probes in genome version 3.0, an additional reciprocal blast with released sequences for all of the probes was employed. If a given gene and probe pair were each other’s top hits in the reciprocal blast results, then the probe was considered to represent the gene. The relative expression levels of all of the *TIFY* genes in xylem, roots, mature and young leaves, male and female catkins were obtained from the *PopGenExpress* database. A heat map for the expression levels of all of the *TIFY* genes in the selected tissues was prepared with Cluster 3.0 software (de Hoon et al., 2004); the dendrogram was visualized using JavaTreeview^6^.

### Plant Material and Stress Treatments

The hybrid poplar variety “NL895” (*P. euramericana*) was used in this study. The female parent of NL895 is *P. deltoids* cv. Lux” (I-69) and the male parent is also a hybrid variety “I-45” (*P. × euramericana*). Tissue culture seedlings of “NL895” were grown in 3-L pots containing a sand-peat (50:50, v/v) mixture, and the pots were placed in a greenhouse under a 16/8 h photoperiod. The light intensity in the greenhouse was set at 12,000 Lx. The temperatures were set at 28 and 25°C for day and night, respectively. The seedlings were watered twice per month with Hoagland’s complete nutrient solution. Additionally, the seedlings were watered with distilled water 2–3 times a week. Seedlings with 6–10 fully expanded leaves were used for the treatments.

For the plant hormone treatments, “NL895” seedlings were treated with 0.2 mM jasmonic acid (JA) and 0.5 mM salicylic acid (SA). Leaves with LPIs (leaf plastochron indices) ranging from 4 to 10 were sprayed with JA or SA solution. To ensure that the hormone treatments were uniform, leaves were sprayed on both sides just until the leaf surfaces began to form small drops. Leaf samples were collected at 0, 2, 6, 12, and 24 h after treatment. Sprayed leaves from each individual poplar plant were pooled into one biological sample. Three replicates were carried out for each treatment.

For the leaf rust pathogen infection treatments, a virulent *M. larici-populina* strain used as fungal material in this study was obtained from *P. simonii* Carr., which showed typical leaf rust symptoms (yellow bubbles on the lower surface of leaf) in the summer of 2015. This strain was also used as the fungal material in another study (Wang et al., 2017). The urediniospores of *M. larici-populina* isolates were allowed to multiply on 1-year-old potted *P. simonii* Carr. cuttings and diluted into an urediniospore suspension in agar-water (0.1% agar in distilled water). The concentrations of the urediniospore suspensions were 1–2 mg/ml. Fully expanded leaves from “NL895” with leaf plastochron indices (LPIs) of 4–10 from each branch were spray-inoculated on their abaxial surfaces with the prepared urediniospore suspension. Leaves from one separate cutting were considered to be one sample. Leaf samples were collected after 0, 2, 4, and 8 days treatments. The samples were immediately frozen in liquid nitrogen and stored at -80°C until required for further analysis.

### Quantitative Real-Time RT-PCR Analysis

Total RNA was isolated using an RNAprep Pure Kit (for Plants) according to the manufacturer’s protocol [TIANGEN Biotech (Beijing) Co., Ltd., Beijing, China]. The quality of all of the RNA samples was examined by performing agarose gel electrophoresis. The first strand cDNA was synthesized using the TransScript One-Step gDNA Removal and cDNA Synthesis SuperMix Kit (TransBionovo Co., Ltd., Beijing, China). The primers used for the qRT-PCR assay of TIFY genes are listed in Supplementary Table S2. PCR products from cDNA or genomic DNA templates using these primer pairs were initially sent for sequencing to confirm that the correct targets had been amplified (data not shown). The correct primer pairs were used for subsequent qRT-PCR assays that were performed on the LightCycler 96 (Roche) platform by using the FastStart Essential DNA Green Master Mix (Roche). For each sample, two reference genes (ACTIN and 18S) were used to standardize the mRNA abundance, and three replications were performed. The 2^-ΔΔCq^ method was used to calculate the relative gene expression based on the qRT-PCR data (Livak and Schmittgen, 2001).

## Results

### Characteristics of TIFY Family Genes in Poplar

**FIGURE 1:**
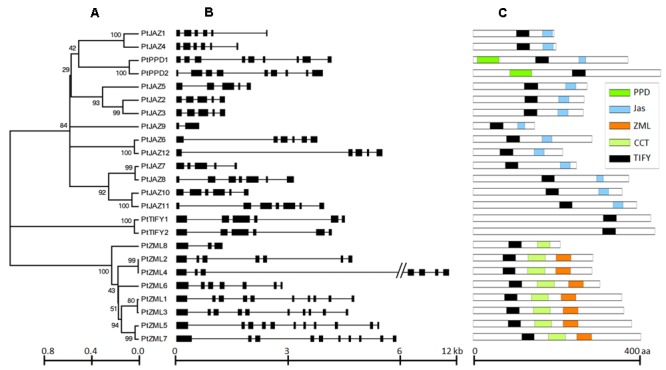
**Phylogeny, structures and domains/motifs of the poplar TIFY gene family.** The numbers labeled on the tree nodes in **(A)**; represent bootstrap values in **(C)**; black box represents exon and line represent intron in **(B)**.

**FIGURE 2:**
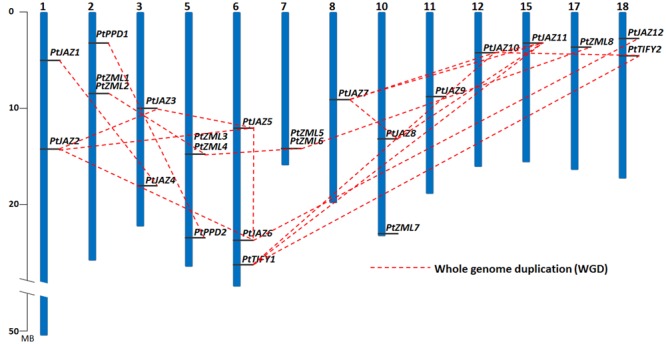
**Chromosomal locations and WGD patterns of the poplar TIFY gene family.** Blue bars represent 19 chromosomes and the gray lines on blue bars represent locations of TIFY genes. A dash line indicate a duplicated gene pair.

A genome-wide search of TIFY family genes in poplar yielded a total of 24 non-redundant genes that were found to harbor the TIFY domain. The 24 non-redundant genes represent a total of 99 transcripts in the *P. trichocarpa* genome annotation version 3.0 (**Table 1**) and all the 99 transcripts have the TIFY domain. To avoid complexity in the subsequent study, only the “0.1” gene model for the 24 non-redundant genes were kept in further analyses. These genes were considered to be TIFY family genes (**Table 1**). Their phylogeny and gene structures are shown in **Figures 1A,B**, respectively. Of these 24 TIFY domain-containing genes, 12 of the predicted proteins contain both the TIFY domain and the Jas motif; these proteins were designated as PtJAZ1 through 12. Seven proteins have both a TIFY domain and CCT and ZML motifs, and one protein has both a TIFY domain and a CCT motif; thus, these eight proteins were designated as PtZML1 through 8. Two proteins contain the TIFY domain and the PPD motif, and they were designated as PtPPD1 and PtPPD2. Note that PtPPD1 also has a Jas motif in its protein structure. The remaining two proteins contain only the TIFY domain, and they were designated as PtTIFY1 and PtTIFY2. All of the domains or motifs located in the 24 TIFY genes are illustrated in **Figure 1C**. The nomenclatures of these genes were designated based on both their subfamilies and their locations on the poplar chromosomes (**Figure 2**). The correspondence of our nomenclatures for the identified TIFY family genes and their original gene IDs released by the *P. trichocarpa* genome annotation version 3.0 are shown in **Table 1**. In previous report, 25 TIFY proteins were identified using the *P. trichocarpa* genome version 1.0 annotations when global comparisons of the gene family were performed in a number of plant species (Bai et al., 2011). When comparing our results with theirs, we found PtZML4 and 5 were corresponding to 3 ZML proteins in Bai‘s results; while the other 22 TIFY genes are consistent in the two identifications.

**Table 1:** TIFY family of genes in poplar.

Gene ID	Gene locus ID	Transcript number	Strand	Start (bp)	End (bp)	ORF (aa)	Duplication
PtJAZ1	Potri.001G062500	2	+	4842780	4845551	196	WGD
PtJAZ2	Potri.001G166200	4	–	13947989	13949828	269	WGD
PtJAZ3	Potri.003G068900	2	+	9718300	9720218	267	WGD
PtJAZ4	Potri.003G165000	3	–	17563038	17565340	201	WGD
PtJAZ5	Potri.006G139400	4	–	11681034	11683548	276	WGD
PtJAZ6	Potri.006G217200	8	+	22993284	22997384	288	WGD
PtJAZ7	Potri.008G133400	12	–	8848283	8851667	250	WGD
PtJAZ8	Potri.010G108200	8	+	12780105	12783705	377	WGD
PtJAZ9	Potri.011G083900	1	–	8532739	8533794	149	N/A
PtJAZ10	Potri.012G044900	10	+	4116344	4120741	361	WGD
PtJAZ11	Potri.015G035800	1	+	3132632	3136562	396	WGD
PtJAZ12	Potri.018G047100	3	+	4408305	4414489	217	WGD
PtZML1	Potri.002G110800	4	–	8198186	8203728	360	WGD
PtZML2	Potri.002G110900	4	–	8206492	8211812	290	Tandem
PtZML3	Potri.005G152500	5	–	14348124	14353280	365	WGD
PtZML4	Potri.005G152800	1	–	14386485	14398559	288	N/A
PtZML5	Potri.007G116500	2	+	13730413	13732003	211	WGD
PtZML6	Potri.007G116700	4	+	13736385	13742259	384	Tandem
PtZML7	Potri.010G251600	1	+	22342132	22345674	307	N/A
PtZML8	Potri.017G042200	5	–	3570422	3576737	406	WGD
PtPPD1	Potri.002G048500	6	–	3161909	3166359	375	WGD
PtPPD2	Potri.005G214300	5	+	22734458	22738357	454	WGD
PtTIFY1	Potri.006G247500	3	+	25449398	25454621	430	WGD
PtTIFY2	Potri.018G033700	1	–	2692940	2697905	440	WGD

According to **Figure 1B**, all of the TIFY family genes have more than one exon. *PtJAZ9* has the smallest number of exons at 2, whereas *PtZML5* has the largest number of exons at 11. The genomic sequences of most of these genes are less than 6 kb in length, but *PtZML4* has the longest genomic sequence of approximately 12 kb. The gene *PtJAZ9* has the shortest open reading frame (ORF) of 149 AA, whereas PtPPD2 has the longest ORF of 454 AA. Insights into the phylogeny and gene structures of genes has indicated that proteins that are closely related phylogenetically tend to have similar gene structures. For example, *PtJAZ1* and *PtJAZ4* have the same number of exons in the same arrangement and tend to cluster together in the phylogenetic tree; this phenomenon was also observed for *PtTIFY1* and *PtTIFY2* and *PtZML1* and *PtZML3*. In order to further insight into the phylogeny of TIFY proteins within plant, TIFY family proteins of three other representative species, including Arabidopsis, rice and grapes, were also employed to construct a phytogenic tree. Clearly, TIFY proteins in the four plants can be grouped into four clades (**Supplementary Figure S1**). According to this figure, proteins in the clade 1 and 2 tend to only have TIFY domain and have ZML motif, respectively; while proteins in the clade 3 and 4 have no clear difference when comparing with their domains or motifs.

The chromosomal locations of these 24 TIFY family genes is shown in **Figure 2** and **Table 1**, and they are found on 13 of the 19 chromosomes. Chromosomes 4, 9, 13, 14, 16, and 19 contain no TIFY genes. Because duplication usually contributes to the expansion of gene families, we investigated the duplication patterns of each of the genes. In total, 19 genes were produced by whole genome duplication (WGD), and two genes were produced by tandem duplication. The other three genes did not show duplication patterns. The two tandemly duplicated genes, *PtZML2* and *Pt ZML6*, were found to be tandemly copied from *PtZML1* and *PtZML5* (**Figure 2**), respectively, whereas both *PtZML1* and *PtZML5* were produced by WGD. This result suggested that *PtZML1* and *PtZML5* were the ancestors of *PtZML2* and *PtZML6*, respectively. The gene duplication patterns for all 24 of the TIFY genes showed that WGD played large roles in this gene family in poplar.

### *In silico* Analysis of Gene Expression Profiles in Different Tissues

**FIGURE 3:**
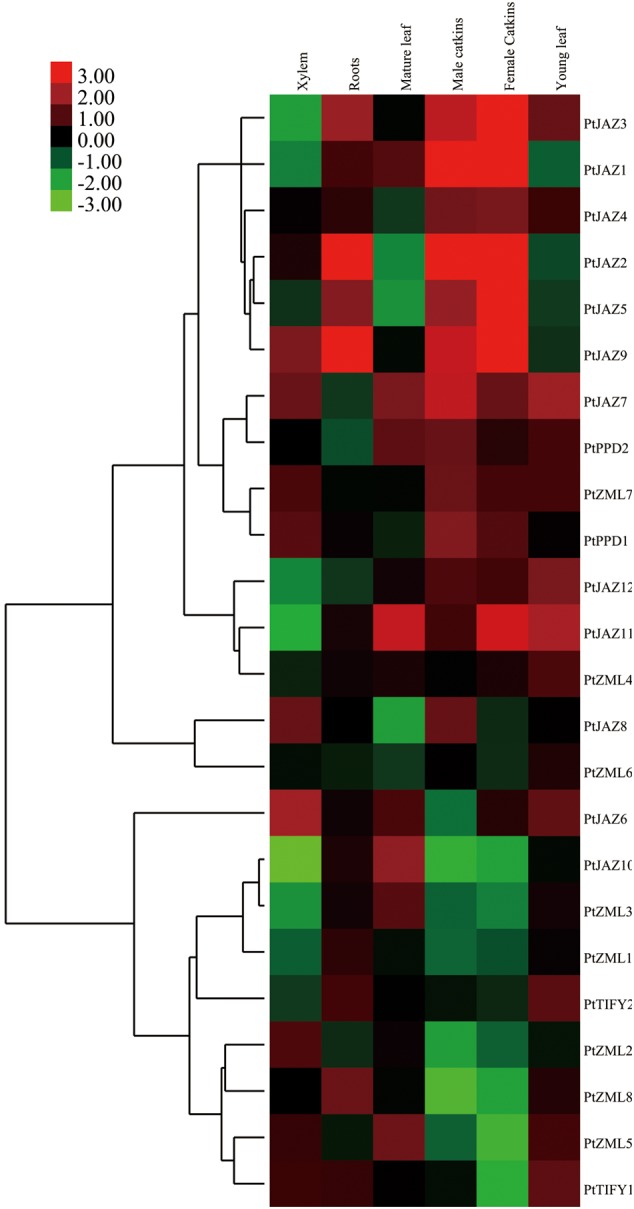
**Hierarchical clustering of the expression profiles of the poplar TIFY gene family across different tissues.** The relative expression levels of all of the *TIFY* genes in xylem, roots, mature and young leaves, male and female catkins were obtained from the *PopGenExpress* database. The expression of a mediate developmental stage of the plant material was set as control and all the gene expression at this stage was considered as “1” in the *PopGenExpress* database. A heat map for the expression levels of all of the *TIFY* genes in the selected tissues was prepared with Cluster 3.0 software.

Since the transcriptional abundance of a gene in different tissues is usually indicative of its function, we investigated the expression profiles of the 24 TIFY family genes in poplar. By analyzing of the publicly available expression data from the *PopGenExpress* database, we were able to obtain the relative expression levels of all 24 TIFY family genes in six tissues: xylem, roots, mature and young leaves and male and female catkins (Supplementary Table S1 and **Figure 3**). All 24 genes showed very high gene expression levels in female (7.11 ± 4.54) and male catkins (5.84 ± 1.63), whereas the lowest expression levels were found in roots (Supplementary Table S1). This result may suggest that the TIFY family of genes plays important roles in poplar catkin development.

Genes that are expressed abundantly in one or several specific tissues usually indicate their function related to the formation of these organs or the corresponding plant development. Further insights into the expression levels for each individual gene indicated that TIFY genes showed extremely high expression levels in some tissues. We filtered these types of genes when their expression was greater than two-fold the average expression level of all TIFY genes in the tissue. In total, seven TIFY genes were preferentially expressed in a given tissue, and some tissues had more than one preferentially expressed gene. *PtJAZ6* and *PtJAZ9* were preferentially expressed in xylem; *PtJAZ2* and *PtJAZ9* were preferentially expressed in roots and mature leaves; *PtJAZ1* and *PtJAZ2* were preferentially expressed in both male and female catkins, and *PtJAZ9* was preferentially expressed in female catkins; *PtJAZ7* and *PtJAZ11* were preferentially expressed in young leaves (Supplementary Table S1 and **Figure 3**). These data suggest that *PtJAZ1, PtJAZ2, PtJAZ6, PtJAZ7*, and *PtJAZ9* could be responsible for the formation of these tissues or contribute to the functions of these tissues. By summarizing these results, we were able to find that a large proportion of genes in the JAZ subfamily were filtered out by our method, although some genes in other subfamilies also showed relatively high abundance in the seven selected tissues. This suggests that most of the JAZ subfamily in the TIFY gene family plays important roles in poplar development. In addition, the expression values for the two reference genes, Actin and 18S, across the five developmental tissues were also obtained and listed in Supplementary Table S1. The data revealed these two genes were expressed similarly in different tissues and it suggested that these two genes were suitable for the subsequent qRT-PCR assay.

### Expression Profiles of Poplar TIFY Genes in Response to JA and SA Treatments

**FIGURE 4:**
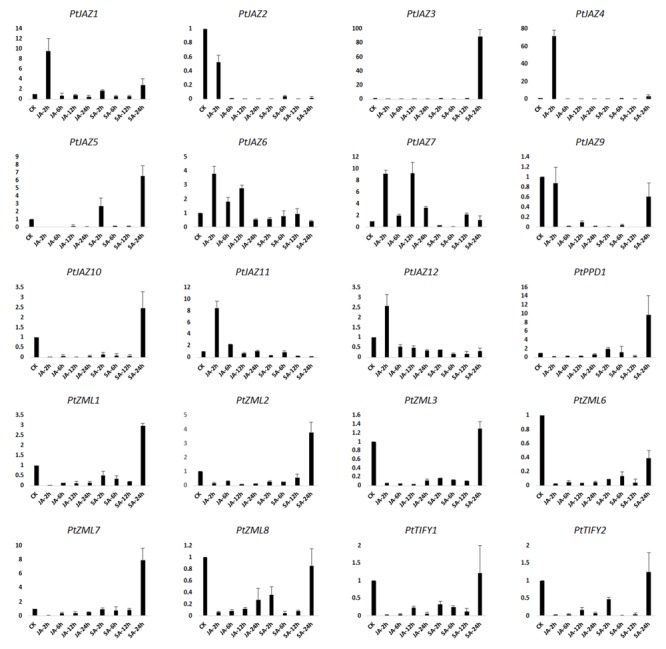
**qRT-PCR assays of the expression profiles of the poplar TIFY gene family genes after MeJA and SA treatments.** CK indicated untreated samples; JA-2 h, JA-6 h, JA-12 h, JA-24 h indicated samples collected from leaves after 2, 6, 12 and 24 h of MeJA treatment, respectively; and SA-2 h, SA-6 h, SA-12 h, and SA-24 h indicated samples collected from leaves after 2, 6, 12, and 24 h of SA treatment, respectively. *Y*-axis indicates relative expression folds when comparison with CK.

JA and SA are hormones that play important roles in signal transduction when plants are challenged with biotic and abiotic stresses. Treating plants with JA, SA or their derivatives MeJA and MeSA can modulate the symptoms of pathogenic or herbivory damage on plants (Yamada et al., 2012; Zhang et al., 2016). To investigate which genes in TIFY gene family can respond to these two plant hormones, we examined the expression profiles of TIFY genes in poplar leaves after treatment with MeJA and SA solutions. The relative expression levels were assayed by qRT-PCR. In the description to convenience, CK indicated untreated samples; JA-2 h, JA-6 h, JA-12 h, JA-24 h indicated samples collected from leaves after 2, 6, 12 and 24 h of MeJA treatment, respectively; and SA-2 h, SA-6 h, SA-12 h, and SA-24 h indicated samples collected from leaves after 2, 6, 12, and 24 h of SA treatment, respectively. In total, we were able to examine the expression profiles for 20 of the 24 TIFY genes; the expression profiles of *PtJAZ8, PtPPD2, PtZML4* and *PtZML5* were not examined due to unsuccessful primer design. The relative expression profiles of all 20 genes for all nine samples are shown in **Figure 4**.

Fold-changes in expression larger than 2.0 or less than 0.5 and also with a *p*-value less than 0.05 between CK and time points after hormone treatment was considered that gene expression was influenced by MeJA or SA treatment. According to the data presented in **Figure 4** and this criterion, gene expression patterns could be classified in four groups. The first group showed that both MeJA and SA increased gene expression at some time points but decreased it at other time points. This group included *PtJAZ1, PtJAZ4* and *PtJAZ7*. The second group showed that both MeJA and SA decreased gene expression at all of the time points (except SA-24 h). This group included *PtJAZ2, PtJAZ9, PtZML3, PtZML6, PtZML8, PtTIFY1* and *PtTIFY2*. Expression of most of the genes in this group was significantly decreased at JA-2 h, JA-6 h, JA-12 h, JA-24 h, SA-2 h, SA-6 h, and SA-12 h. In the third group, MeJA decreased gene expression at most time points, whereas SA increased it at some time points and decreased it at others. This group included *PtJAZ3, PtJAZ5, PtJAZ10, PtPPD1, PtZML1, PtZML2* and *PtZML7*. In the fourth group, MeJA increased gene expression at some time points and decreased gene expression at the other time points, whereas SA decreased gene expression at all of the time points. This group included *PtJAZ6, PtJAZ11* and *PtJAZ12*.

### Expression Profiles of Poplar TIFY Genes in Response to the Poplar Leaf Rust Pathogen *M. larici-populina*

Previous reports suggested JAZ protein might act as TFs in response to rust fungi (Duplessis et al., 2009). To identify TIFY genes that respond to poplar leaf rust disease, we collected leaf samples from the poplar hybrid variety “NL895” that had been infected with *M. larici-populina* (see Materials and Methods); these samples were designated as CK, PI2, PI4, and PI8. The CK represented samples collected immediately after leaf rust infection, while PI2, PI4, and PI8 represented samples collected from leaves treated by urediniospore suspension of *M. larici-populina* after 2, 4, and 8 days, respectively. The symptom of leaf rust on leaves of “NL895” at these four selected time points were different. No visible symptom was observed at CK and 2 dpi; while very small white milk-white and plenty of uredinia could be observed at 4 and 8 dpi, respectively (data not shown). Previously, 48 hpi (equal to 2 dpi) was reported to be a key time point for the rust development (Rinaldi et al., 2007). Therefore, our plant material could be able to represent all key developmental stages of *M. larici-populina* on poplar leaves.

**FIGURE 5:**
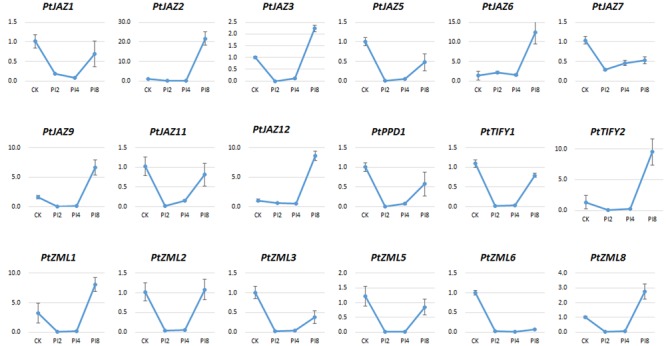
**qRT-PCR assays of the expression profiles of the poplar TIFY gene family in response to *M. larici-populina*.** The hybrid poplar variety “NL895” was used as plant material. CK indicated the control samples; 2, 4, and 8 dpi indicated samples collected from “NL895” leaves after 2, 4, and 8 days treatment by *M. larici-populina* inoculation. The virulent *M. larici-populina* strain used as fungal material in this qRT-PCR assay (see Materials and Methods). *Y*-axis indicates relative expression folds when comparison with CK.

The relative expression of genes for PI2, PI4, and PI8 versus CK for all of the 24 TIFY genes was determined by qRT-PCR assays. We were able to test the expression of 18 of the 24 TIFY genes (**Figure 5**). According to the expression profiles of these 18 TIFY genes and using the criterion of fold changes less than 0.5 or larger than 2 and also with a *p*-value less than 0.05 when comparing CK with the other three treatments, we were able to classify them into four groups. The first group showed that gene expression was reduced by *M. larici-populina* infection at all the three stages of PI2, PI4, and PI8 when comparing with CK. This group included *PtJAZ5, PtJAZ7, PtZML3, PtZML5* and *PtZML6*. The second group showed similar gene expression level of CK and PI8, while gene expression was largely reduced at PI2 and PI4 stages. This group had five genes and it included *PtJAZ1, PtJAZ11, PtPPD1, PtTIFY1* and *PtZML2*. The third group showed that gene expression was similar at CK, PI2 and PI4 and highly increased at PI8. This group had six genes and it included *PtJAZ2, PtJAZ6, PtJAZ9, PtJAZ12, PtTIFY2* and *PtZML1*. The fourth group showed that gene expression was reduced at PI2 and PI4 and highly increased at PI8. This group had two genes and it included *PtJAZ3* and *PtZML8*. Based on these data, we could conclude that all tested TIFY genes respond to *M. larici-populina* infection and they also showed different responsive patterns.

## Discussion

In this study, we were able to identify 24 TIFY genes in the poplar genome using the *P. trichocarpa* genome annotation version 3.0. However, the similar total number of TIFY genes between the two identifications could suggest the accuracy of our results. In other plants (e.g., Arabidopsis, rice, apple, soybean, and grape), the number of TIFY genes ranges from 18 to 34 (Ye et al., 2009; Bai et al., 2011; Zhang et al., 2012; Li et al., 2014; Sirhindi et al., 2016). This result suggested that the number of TIFY genes in poplar is within the normal range. In a number of previous studies, the expansion of a given gene family usually indicates preferential roles that they played during the plant’s development. A largely expanded gene family in a plant genome sometimes results in unique characteristics for the plant. For example, when the genomes of *P. trichocarpa* and *P. euphratica* were compared, several gene families likely to be involved in tolerance to salt stress were found to be present in significantly greater gene copy numbers within the *P. euphratica* lineage (Ma et al., 2013). The expansion of TIFY genes in most plants did not show clear differences according to the information we obtained; this phenomenon might indicate that the function of TIFY genes in plants is essential and that they are involved in the basic plant processes.

The duplication pattern of a gene usually reveals how the gene was generated, how its function evolved and what roles it may played in plant growth and development (Wang et al., 2013). WGD is predicted to produce new genomic regions based on an ancestral genome, and this event occurs once or several times over many (sometimes hundreds of) millions of years (Wang et al., 2011). After WGD, the functions of the newly produced genes are usually modified to reduce genomic redundancy and could increase the plant’s ability to adapt to various growth environments (Flagel and Wendel, 2009; Magadum et al., 2013). In other words, gene families that were chiefly produced by WGD usually existed in the “ancestral genome” before duplication. This indicates that the functions of members of a gene family are indispensable for plant growth and development in a given species. In addition, genes in families that were mainly produced by WGD usually evolve slowly (Wang et al., 2013; Soltis and Soltis, 2016). In this study, we found that 19 of the 24 *TIFY* genes were produced by WGD. This revealed that WGD made a large contribution to the generation of the TIFY gene family in poplar and this result is consistent with reports from the poplar genome sequencing project (Tuskan et al., 2006). It also suggested that the TIFY genes are indispensable for poplar growth and development; this prediction is consistent with the prediction from gene numbers in TIFY families of most of the plants investigated.

Since JA and SA are important hormones for signal transduction when plants encounter pathogens, we chose to investigate whether there was commonality between the TIFY gene expression profiles in poplar leaves after JA/SA treatments and leaf rust pathogen infection. TIFY family genes could be arranged into four groups both after JA/SA treatments and leaf rust pathogen infection. However, we were not able to see a clear rule (data not shown) after analyzing the overlapping TIFY genes between the two classifications. This result suggests that although most of the TIFY genes can respond to a single elicitor, JA or SA treatment and leaf rust pathogen infection, the mechanisms underlying the response of the TIFY genes to each elicitor are different. This also indicates that gene regulation and signal transduction during plant responses to biotic stress are complicated.

Although there was no rule for the TIFY gene expression profiles in poplar leaves between JA/SA treatments and leaf rust pathogen infection, we were still able to obtain some valuable information by analyzing their expression profiles after phytohormone treatments and leaf rust pathogen infection in poplar. First, a large proportion of TIFY family genes can respond to JA/SA treatments and leaf rust pathogen infection. The expressions of all 20 tested TIFY genes were strongly influenced by JA/SA treatments (**Figure 4**), whereas all 18 tested TIFY genes were strongly influenced by leaf rust pathogen infection. Because TIFY family genes were reported to function in signal transduction when plants encountered various stresses and 65% of its members were found to respond to leaf rust pathogen infection, we speculated that the TIFY gene family in poplar also plays important roles in deploying the defense system against leaf rust via the JA/SA signal transduction pathway. Second, the TIFY genes which showed large changes in expression upon leaf rust pathogen infection could be used as a potential resource to increase resistance to the disease caused by *M. larici-populina*. The available transgenic platform that has been created in poplar allows overexpression or reduced expression transgenic lines to be generated. For example, the expressions of *PtJAZ3, PtJAZ6, PtJAZ9*, and *PtJAZ15* were largely increased after leaf rust pathogen infection, and we can generate transgenic poplar lines that overexpress these genes (**Figure 5**). In contrast, the expressions of *PtJAZ5, PtJAZ7, PtZML3, PtZML5* and *PtZML6* were largely reduced after leaf rust pathogen infection, and we can generate transgenic poplar lines with reduced expression of these genes (**Figure 5**). Third, several previous studies showed a number of other genes could be able to respond to leaf rust infection and these genes would play important roles in defensive regulation in polar. For instance, a small number of pathogen-defense genes encoding PR-1, chitinases, and other pathogenesis-related proteins were found to be consistently upregulated throughout the whole leaf rust infection (Miranda et al., 2007); while 1,730 and 416 significantly differentially expressed transcripts were also found in the incompatible interaction between poplar and poplar rust (Rinaldi et al., 2007; Azaiez et al., 2009). Combining the previous reports with our study, we might be able to speculate that TIFY genes showed response to leaf rust infection could interact with genes reported in these studies. Finally, although TIFY family genes have been reported to function in signal transduction when plants encounter various stresses, the detailed mechanisms of how they perform their functions are still not clear, especially in the woody perennial plant poplar. Therefore, more work to understand how TIFY genes perform their functions in poplar should be performed in the future.

In summary, we conducted a genome-wide identification of TIFY genes in poplar. The characteristics of this gene family was carefully investigated. We also analyzed their expression profiles in response to phytohormone treatments and *Melampsora larici-populina* infection in poplar. The results of this study have provided valuable information for further functional characterisation of TIFY genes in poplar and have also provided candidate genes for the improvement of disease resistance in poplar.

## Author Contributions

NW, WX, HY, and PC organised the entire project and performed the experiments and data analysis. NW and JL wrote and edited this manuscript.

## Conflict of Interest Statement

The authors declare that the research was conducted in the absence of any commercial or financial relationships that could be construed as a potential conflict of interest.

## Footnotes

1 http://popgenie.org/

2 http://pfam.xfam.org/

3 https://phytozome.jgi.doe.gov/pz/portal.html

4 https://phytozome.jgi.doe.gov/pz/portal.html

5 http://www.bar.utoronto.ca/

6 https://sourceforge.net/projects/jtreeview/

## Supplementary Material

FIGURE S1 **Phylogeny of the TIFY proteins in the four representative species.** The protein names start with “At” indicate TIFY genes from Arabidopsis; the protein names start with “Os” indicate TIFY genes from rice; the protein names start with “Vv” indicate TIFY genes from grapes; the protein names start with “Pt” indicate TIFY genes from poplar.
