# Supplementary figures and images for: Identification of TIFY Family Genes and Analysis of Their Expression Profiles in Response to Phytohormone Treatments and Melampsora larici-populina Infection in Poplar
Source: Front Plant Sci. 2017 Apr 5;8:493. doi: 10.3389/fpls.2017.00493 (PMC5380741; doi:10.3389/fpls.2017.00493)

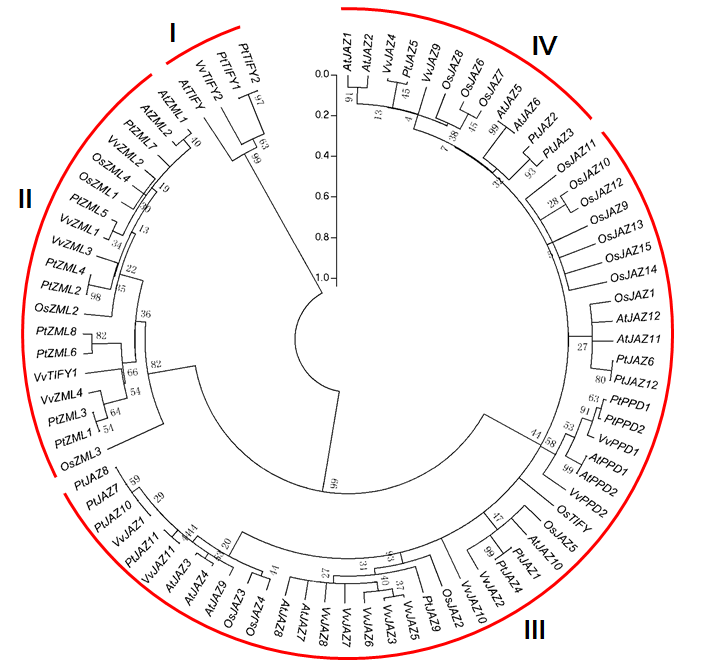

Supplement: FIGURE S1 — Phylogeny of the TIFY proteins in the four representative species. The protein names start with “At” indicate TIFY genes from Arabidopsis; the protein names start with “Os” indicate TIFY genes from rice; the protein names start with “Vv” indicate TIFY genes from grapes; the protein names start with “Pt” indicate TIFY genes from poplar. [file Image_1.TIF]
